# Mild Exercise Rescues Steroidogenesis and Spermatogenesis in Rats Submitted to Food Withdrawal

**DOI:** 10.3389/fendo.2020.00302

**Published:** 2020-05-13

**Authors:** Alessandra Santillo, Antonia Giacco, Sara Falvo, Federica Di Giacomo Russo, Rosalba Senese, Maria Maddalena Di Fiore, Gabriella Chieffi Baccari, Antonia Lanni, Pieter de Lange

**Affiliations:** ^1^Department of Environmental, Biological, and Pharmaceutical Sciences and Technologies, University of Campania “L. Vanvitelli, ” Caserta, Italy; ^2^Department of Science and Technology, University of Sannio, Benevento, Italy

**Keywords:** testis, StAR, 3β-hydroxysteroid dehydrogenase, P450 aromatase, steroidogenesis, spermatogenesis, food withdrawal, exercise

## Abstract

The present investigation was undertaken to increase our insight into the molecular basis of the physiological changes in rat testis induced by food withdrawal, and to clarify whether reduced testicular function can be ameliorated by mild exercise. Male rats were selected for four separate experiments. The first of each group was chow-fed, the second was chow-fed and submitted to exercise (5 bouts in total for 30 min at 15 m/min, and 0° inclination), the third was submitted to food withdrawal (66 h) and the fourth was submitted to food withdrawal and to exercise. At the end of experiments, we investigated (i) serum and testicular sex hormone levels; (ii) protein levels of StAR, 3β-Hydroxysteroid dehydrogenase (3β-HSD) and P450 aromatase, which play a key role in steroid hormone biosynthesis; and (iii) protein levels of mitotic and meiotic markers of spermatogenesis in rats, in relation to testis morphology and morphometry. We found that mild exercise or food withdrawal alone induced a significant increase or decrease in both serum and testis testosterone levels, respectively. Interestingly, we found that these levels were brought back to basal levels when food withdrawal was combined with mild exercise. The changes in testosterone levels observed in our experimental groups correlated well with the expression of steroidogenic enzymes as well as with spermatogenic activity. With mild exercise the increased testosterone/17β-estradiol (T/E_2_) ratio in the testis correlated with an increased spermatogenic activity. The T/E_2_ ratio dropped in fasted rats and was significantly reversed when food withdrawal was combined with exercise. Histological and morphometric analyses confirmed that spermatogenic activity varied in concomitance with each experimental condition. Importantly, the testis and serum T/E_2_ ratios correlated, confirming that exercise rescues the decline in food withdrawal-induced spermatogenesis. In conclusion, this study highlights that mild exercise normalizes the reduced spermatogenic activity caused by food withdrawal through the modulation of the steroidogenic pathway and restoring the T/E_2_ ratio, underlining the beneficial effects of mild exercise on the prevention and/or amelioration of reduced testis function caused by restricted caloric intake.

## Introduction

Testis functional impairment is worsened in Western society leading to decreased fertility rates (1). Lifestyle-related causes of reduced testicular function include factors such as food intake. For example, overnutrition relates to testicular malfunction, highlighted by decreased free testosterone serum levels, due to decreased gonadotropin levels, resulting in impaired spermatogenesis (2). Men having a body mass index (BMI) of over 25, compared with those exhibiting a normal BMI, are indeed reported to have an average reduction in sperm number and motility of 25% (3). Exercise training has been recommended in these subjects to reduce body mass (4). However, although exercise training is increasingly being promoted by physicians as one of the main life style interventions for primary prevention of obesity-related diseases (5–10), data connecting exercise training with testicular function are conflicting: indeed, high-intensity exercise reduces testicular function, and data concerning moderate exercise training are controversial (1).

Although weight loss is warranted in overweight men with fertility problems, a great deal of investigations have verified that reduced food intake relates to reduced testicular steroidogenesis and spermatogenesis resulting in a dramatic reduction of reproductive functions which is related to decreased secretion of pituitary hormones such as luteinizing hormone (LH) and prolactin leading to reduced serum testosterone levels (11, 12). In particular, in rats after 7 days of food withdrawal, levels of the aforementioned hormones become rate-limiting for efficient steroidogenesis (11). It is known, furthermore, that food withdrawal, diets with low protein, or with restricted calories lead to impaired gonadal function (13–15).

The present study was carried out in order to obtain further evidence for impaired testis function during food withdrawal in the rat, and to elucidate whether this can be modified by mild exercise, studying the separate effects of food withdrawal and exercise, as well as that of their combination, on steroidogenesis and spermatogenesis. The experiments were performed at thermoneutrality in order to produce data that are reminiscent to similar data obtained in humans (16, 17).

We investigated (i) testicular compared to serum sex hormone levels; (ii) testicular protein levels of Steroidogenic acute regulatory protein (StAR), 3β-Hydroxysteroid dehydrogenase (3β-HSD), and P450 aromatase, which play a key role in steroid hormone biosynthetic pathway; (iii) testicular protein levels of mitotic and meiotic markers of spermatogenesis; and (iv) histological and morphometric parameters in rat testis.

## Materials and Methods

### Animals

This animal study followed the current guidelines of the European Commission for the care and use of laboratory animals. The project was approved by the Ethics Committee for Animal Experiments of the University of Campania “Luigi Vanvitelli” and the Italian Ministry of Health (authorization 704/2016 PR pursuant to article 31 of legislative decree 26/2014). Male Wistar rats (*n* =16, 3 months old, initial weight around 300 g) were housed separately at thermoneutrality (28°C) with *ad-libitum* access to water and chow [fat content (mg/kg): palmitic acid 4387; palmitoleic acid 202; stearic acid 675; oleic acid 5046; linoleic acid 12335; linoenic acid 1169. Energy percentage (metabolizable): carbohydrates 60.4; proteins 29; fat 10.6 J/J; 15.88 KJ gross energy/g. The chow diet was from Muscedola s.r.l., Milan, Italy]. Exercise experiments were carried out as described (17). Briefly, all animals were familiarized with the Panlab treadmill (Harvard Apparatus, Holliston, MA, USA) in order to correct for stress responses related to the environment. Four groups of animals were selected for 4 separate experiments. The first of each group was chow-fed (C), the second was chow-fed and submitted to exercise (E), the third was submitted to food withdrawal (F) and the fourth was submitted to food withdrawal and to exercise (FE). The animals had *ad-libitum* access to water throughout. The exercised animals carried out 5 low intensity treadmill runs (twice daily, 30 min, 15 m/min, 0° inclination. The duration of the experiment was 3 days (66 h from the start of food withdrawal to sacrifice), on the third day the animals were submitted to only one exercise bout. The timespan from the finish of the exercise bout to sacrifice was 4 h. Serum was prepared and stored at −20°C. Testes were dissected out and in part transferred to Bouin's fluid (Sigma Aldrich, Milan, Italy), being subsequently embedded in paraffin for histological analysis, the remainder being stored at −80°C.

### Sex Hormone Steroid Assays in Both Serum and Testis

Testosterone and 17β-estradiol levels in rat serum and testes were determined essentially as described (18). Briefly, samples of 4 animals per experimental group were collected and three replicates for each sample ran in the same ELISA (19).

### Protein Extraction and Western Blot Analysis

To analyze StAR protein levels, mitochondrial fractions of testes were prepared as described (20, 21). Cytosolic fractions were used to assay 3β-HSD, P450 aromatase, PCNA, Aurora B, SYCP3, and β-actin protein levels. Protein concentrations of mitochondrial and cytosolic fractions assessed as described (22). For Western blot analysis, the following primary antibodies were used: StAR polyclonal antibody (assayed on mitochondrial fractions), raised in rabbit, diluted 1:5000 (Elabscience Biotechnology Inc, Houston, Texas), 3β-HSD polyclonal antibody, raised in rabbit, diluted 1:1000 (Elabscience Biotechnology Inc, Houston, Texas); P450 aromatase polyclonal antibody, raised in rabbit, diluted 1:4000 (Santa Cruz Biotechnology, Inc., Santa Cruz, CA); PCNA monoclonal antibody, raised in mouse, diluted 1:1000 (Sigma Aldrich, Inc., St. Louis, MO); Aurora B polyclonal antibody, raised in rabbit, diluted 1:500 (Elabscience Biotechnology Inc, Houston, Texas); SYCP3 polyclonal antibody, raised in mouse, diluted 1:250 (Santa Cruz Biotechnology, Inc., Santa Cruz, CA); β-actin polyclonal antibody, raised in mouse, diluted 1:2000 (Santa Cruz Biotechnology, Inc., Santa Cruz, CA). After washing with TBS-tween, membranes were incubated with horseradish-peroxidase anti-rabbit IgG or anti-mouse IgG secondary antibodies for 1 h at room temperature, followed by signal detection using enhanced chemiluminescence (ECL) (Amersham Bioscience, UK). The amount of proteins was quantified using Image J software (National Institutes of Health, Bethesda, USA) and normalized with respect to β-actin protein. SYCP3 showed as two proteins with MW 33 and 30 kDa; we evaluated the density of both bands and calculated the mean value.

### Histological and Morphometric Analyses

Briefly, 5 μm-thick testis paraffin sections were stained and viewed under a Leica light microscope equipped with a digital camera (Leica ICC50 HD) as described (23) to determine diameters of the seminiferous tubules and lumen, as well as germinal epithelium height as measured with Image J software (NIH, Bethesda, USA).

### Statistical Analysis

The number of animals used for each experimental condition was assessed using a power test as described (17). Two-way analysis of variance (ANOVA, *post-hoc* test: Bonferroni) was performed by using Prism 5.0 (Graphpad, San Diego, CA, USA) to assess significant changes between experimental groups. Data were normally distributed. Differences were considered statistically significant at *P* < 0.05. All data are expressed as means ± standard deviation (SD).

## Results

### Sex Hormone Levels in Serum and Testis

Both serum and testicular testosterone levels increased significantly following exercise with respect to chow-fed controls, whereas food withdrawal induced a significant decrease in testosterone levels compared to both exercise and control groups (Table 1). When food withdrawal was combined with exercise, testosterone levels were significantly induced in respect to those observed with food withdrawal alone, reaching levels comparable to chow fed controls (Table 1).

**Table 1:** Testosterone (T), 17β-estradiol (E_2_) levels and ratio T/E_2_ in rat serum and testis.

	**C**	**E**	**F**	**FE**
**Serum hormone levels**				
Testosterone (ng/ml)	3.8 ± 0.9	11.4 ± 1.9^**^	0.9 ± 0.1^*^^,^ ^a^	2.5 ± 0.5^a^^,^ ^b^
17β-estradiol (pg/ml)	7.3 ± 2.0	8.3 ± 2.5	4.5 ± 0.7	5.2 ± 0.41
Ratio T/E_2_	0.52 ± 0.13	1.37 ± 0.28^**^	0.20 ± 0.02^*^^,^ ^a^	0.48 ± 0.11 ^a^^,^ ^b^
**Testis hormone levels**				
Testosterone (ng/g tissue)	20.9 ± 8.6	85.4 ± 12.3^**^	3.0 ± 2.2^*^^,^^a^	18.0 ± 8.9 ^a, b^
17β-estradiol (pg/g tissue)	76.6 ± 13.0	91.5 ± 20.1	128.9 ± 16.1^*^	106.7 ± 18.3
Ratio T/E_2_	0.27 ± 0.11	0.93 ± 0.084^**^	0.02 ± 0.01^*^^,^^a^	0.17 ± 0.076 ^a, b^

* P < 0.05 vs. C;

** P < 0.01 vs. C;

a P < 0.05 vs. E;

b *P < 0.05 vs. F. C, chow-fed; E, chow-fed submitted to exercise; F, food withdrawal; FE, food withdrawal submitted to exercise (N = 4)*.

In addition, we observed a significant increase in testis 17β-estradiol levels with food withdrawal with respect to control groups (Table 1). Mild exercise alone did not influence 17β-estradiol levels but when combined with food withdrawal, mild exercise restored 17β-estradiol levels to chow-fed controls (Table 1). In serum, no significant changes were observed in 17β-estradiol levels among the experimental groups (Table 1). In both serum and testis, T/E_2_ ratios were significantly increased with exercise alone, decreased with food withdrawal and restored when combined with exercise (Table 1).

### StAR, 3β-HSD and P450 Aromatase Protein Levels

**Figure 1:**
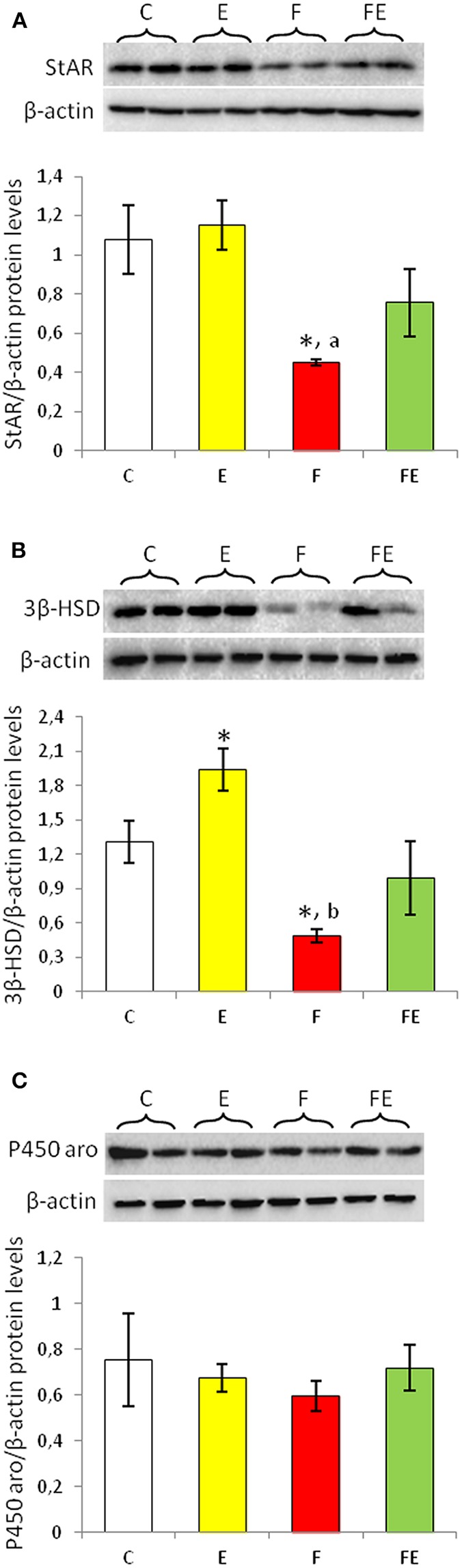
StAR **(A)**, 3β-HSD **(B)**, and P450 aromatase **(C)** Western blot detections (see upper panels) in rat testes. The results are representative of those obtained in each animal (*N* = 4). The lower panels show the amount of proteins quantified using the ImageJ program and normalized with respect to β-actin protein. Values shown represent the means ± S.D. of four animals (two bands shown in upper panels). *P < 0.05 vs. control; ^a^*P* < 0.05 vs. exercise; ^b^ *P* < 0.01 vs. exercise. C, chow-fed; E, chow-fed submitted to exercise; F, food withdrawal; FE, food withdrawal submitted to exercise.

StAR protein levels in rat testis did not change with exercise, whereas its levels were significantly decreased by food withdrawal compared to both chow-fed and exercise groups (Figure 1A). When food withdrawal was combined with exercise, StAR protein levels did not significantly change compared to chow fed group (Figure 1A).

Exercise significantly increased protein levels of 3β-HSD (Figure 1B). 3β-HSD protein levels significantly decreased with food withdrawal compared to both chow-fed and exercise groups, whereas it was comparable to basal value with food withdrawal/exercise (Figure 1B).

Protein levels of P450 aromatase did not change significantly among the experimental groups (Figure 1C).

### PCNA and Aurora B Protein Levels

**Figure 2:**
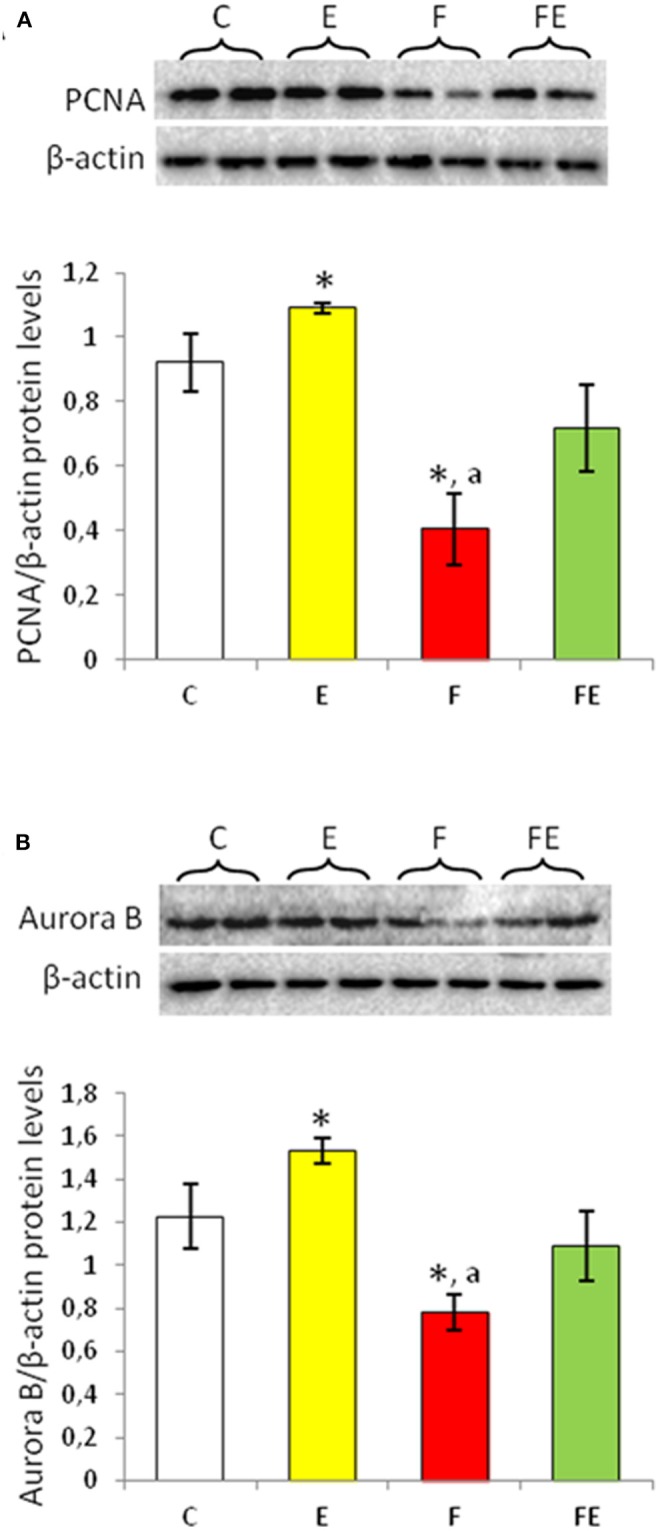
PCNA **(A)** and Aurora B **(B)** Western blot detection (see upper panels) in rat testes. The results are representative of those obtained in each animal (*N* = 4). The lower panels show the amount of proteins quantified using the ImageJ program and normalized with respect to β-actin protein. Values represent the mean ± SD of four animals (two bands shown in upper panels). *P < 0.05 vs. controls; ^a^*P* < 0.05 vs. exercise. C, chow-fed; E, chow-fed submitted to exercise; F, food withdrawal; FE, food withdrawal submitted to exercise.

Proliferating cellular nuclear antigen (PCNA) is a nuclear-expressed protein during the S phase (24). Exercise significantly increased protein levels of PCNA. Its levels significantly decreased with food withdrawal compared to both chow-fed and exercise groups. PCNA protein levels returned to basal value with food withdrawal/exercise (Figure 2A).

Aurora B is involved in the maintenance of genome stability during mitosis and regulates G2-M transition (25, 26). Chow-fed exercise significantly increased Aurora B protein levels compared to the chow-fed controls (Figure 2B). Food withdrawal condition induced a significant decrease in Aurora B protein levels compared to both chow-fed and exercise groups (Figure 2B). In response to food withdrawal/exercise, Aurora B protein levels were close to chow-fed control levels (Figure 2B).

### SYCP3 Protein Levels

**Figure 3:**
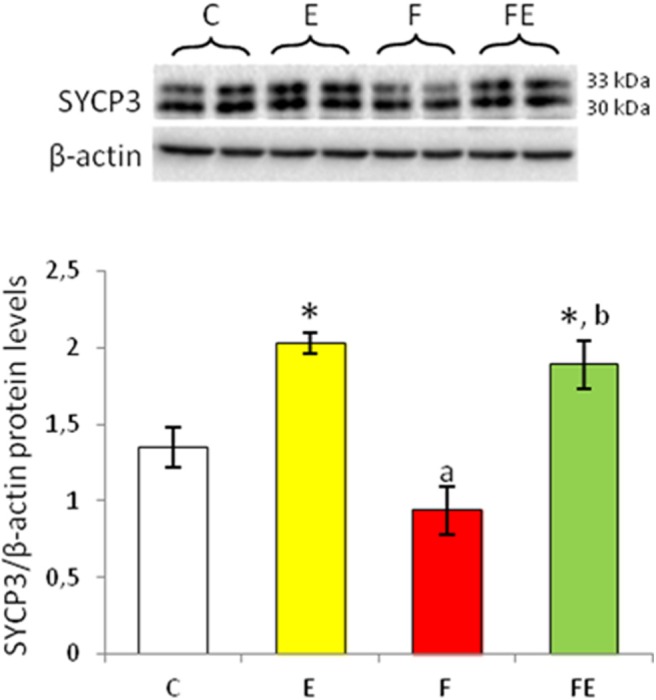
SYCP3 Western blot detection (see upper panels) in rat testes. The results are representative of those obtained in each animal (*N* = 4). The lower panels show the amount of proteins quantified using the ImageJ program and normalized with respect to β-actin protein. Values represent the mean ± SD of four animals (two bands shown in upper panels). *P < 0.05 vs. controls; ^a^*P* < 0.05 vs. exercise; ^b^*P* < 0.05 vs. food withdrawal. C, chow-fed; E, chow-fed submitted to exercise; F, food withdrawal; FE, food withdrawal submitted to exercise.

SYCP3 is expressed at the meiotic prophase encoding synaptonemal complex proteins; it is localized in the lateral elements (27). After exercise, SYCP3 protein levels were significantly induced in both fed and food deprived groups with respect to chow-fed control (Figure 3). Food withdrawal reduced SYCP3 protein levels with respect to exercise and food withdrawal/exercise but not to the control group (Figure 3).

### Histological and Morphometric Analyses

**Figure 4:**
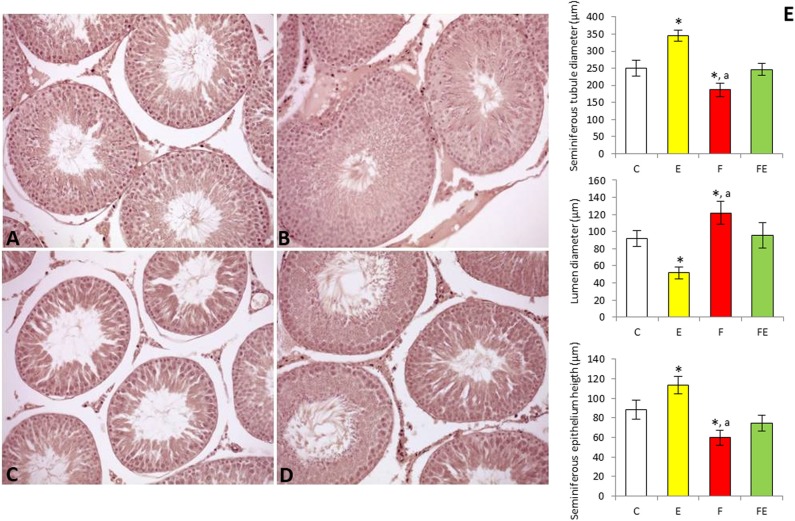
Paraffin sections of the testes from chow-fed **(A)**, exercise **(B)**, food withdrawal **(C)** and food withdrawal/exercise **(D)** rats. Haematoxylin–eosin stain, 200X. Testis morphometric parameters were shown in the graphs **(E)**. *P < 0.05 vs. control; ^a^*P* < 0.05 vs. exercise. C, chow-fed; E, chow-fed submitted to exercise; F, food withdrawal; FE, food withdrawal submitted to exercise.

Histological and morphometric evaluations of the test is supported the effects of the different experimental interventions on spermatogenesis. Control animals exhibited regular testicular morphology, the lumen of the seminiferous tubules being filled with spermatozoa (Figure 4A). Seminiferous tubules of exercised animals had a wider diameter than those of the controls, as well as more reduced lumen and a thicker germinal epithelium (Figure 4E), indicative of an increase in spermatogenic activity (Figure 4B). Testes of rats submitted to food withdrawal showed seminiferous tubules with a smaller diameter compared to both exercise and control groups (Figure 4C), as well as wider lumen and a thinner germinal epithelium (Figure 4E). In this group, the amount of spermatozoa in the lumen was strongly reduced (Figure 4C). The testis of rats submitted to food withdrawal and exercise maintained the morphological characteristics of the controls (Figure 4D), and morphometric parameter values were close to those of the chow-fed group (Figure 4E).

## Discussion

Although weight loss is warranted in overweight men with fertility problems, a great deal of investigations has verified that reduced food intake is associated with a decrease of testosterone levels, which in turn impairs spermatogenesis (28–30). Animal model-based studies have been performed in order to understand the molecular framework of the physiological changes in testis fasted men (14).

In this study, in rats housed at thermoneutrality, we provide evidence of the effects of mild exercise and food withdrawal on sex hormone levels, protein levels of steroidogenic enzymes and both mitotic and meiotic markers of spermatogenesis. Furthermore, we studied whether mild exercise alone or in combination with food withdrawal may improve spermatogenesis.

We present here for the first time that both serum and testis testosterone levels, reduced by food withdrawal, were normalized to control levels in response to mild exercise. The observed changes in testosterone levels may be explained by the change in the testicular protein levels of StAR, a testicular protein necessary for biosynthesis of testosterone, and 3β-HSD, a key enzyme for testicular androgenesis (31–33). Steroid hormone biosynthesis starts with cholesterol store trafficking to the mitochondria, followed by conversion to pregnenolone via P450 side chain cleavage activity at the inner mitochondrial membrane (33–36). Cholesterol transfer between membranes is the rate-limiting step in testosterone biosynthesis, which is facilitated by StAR (32, 34–38). Indeed, it was previously demonstrated that the levels of StAR protein in Leydig cells significantly affects testosterone production (32, 39, 40). Accordingly, we found that when testicular StAR protein levels were reduced, as occurred during food withdrawal, testicular androgenesis is attenuated; when food withdrawal was combined with exercise, testicular StAR protein as well as testis testosterone levels were comparable to controls. The lack of correlation between StAR protein expression and testicular testosterone levels observed in food withdrawal/exercise group compared to food withdrawal group as well as in fed rats subjected to exercise compared to fed rats could be due to an increase in protein activity. On the contrary, rats subjected to intensive exercise have been reported to show decreased serum and testis testosterone levels as well as reduced StAR protein levels (33). Therefore, while high-intensity exercise negatively impacts upon testicular function, our findings provided further evidence that a positive correlation between moderate-intensity exercise and testis function parameters exists.

Interestingly, we found that the changes in testosterone levels in both serum and testis correlated well with 3β-HSD protein levels among the experimental groups. In particular, during the exercise alone when the testosterone levels were higher compared to control rats we found in parallel a significant increase in testis 3β-HSD protein levels. To our knowledge, these findings demonstrated for the first time that mild exercise could influence protein levels of 3β-HSD within the testis.

Conversely, during food withdrawal, when the testosterone levels were low, we observed a concomitant decrease in 3β-HSD protein levels, providing additional evidence that food withdrawal induces inadequate steroidogenesis reducing protein levels of 3β-HSD. Accordingly, Fanjul and Ruiz de Galarreta (1981) (13) demonstrated that starvation reduces serum testosterone by decreasing 3β-HSD activity. Importantly, 3β-HSD protein levels were maintained close to control levels in food withdrawal/exercise group when also testosterone levels resulted unchanged with respect to chow-fed controls, suggesting that mild exercise could be able to improve steroidogenesis in 66 h-fasted rats. However, 3β-HSD protein expression levels in food withdrawal/exercise group compared to food withdrawal group did not correlate with testis testosterone levels. This could be due to an increase in protein activity resulting in the increase of testosterone synthesis.

Testosterone influences spermatogenesis inducing spermatogonial proliferation and meiotic process leading to the production of spermatozoa (41). We found that protein levels of PCNA and Aurora B, two mitotic markers, and SYCP3, a meiotic marker, in testis from mild exercise, food withdrawal and food withdrawal/exercise groups correlated with the steroidogenic pathway protein and testosterone levels in the same groups. These results suggest that exercise and food withdrawal separately induce an increase or decrease in spermatogenesis process, respectively. Moreover, mild exercise was able to restore spermatogenesis activity in food deprived rats, supporting the hypothesis of its beneficial effects on testicular function. Histological and morphometric analyses presented herein confirm these findings. The protein levels of the mitotic and meiotic markers found in both exercise and food withdrawal groups compared to control group suggest an increase both in spermatogonial proliferation and differentiation to spermatocytes in exercise group, while both processes were reduced in food withdrawal group. In food withdrawal/exercise group the protein levels of mitotic markers were not statistically different from both control and food withdrawal groups, whereas protein levels of SYCP3 were significantly higher than in control and food withdrawal groups. An increased differentiation to spermatocytes but constant spermatogonial proliferation in the food withdrawal/exercise group could occur.

It is well-known that once synthetized, testosterone is in part converted by the enzyme P450 aromatase into 17β-estradiol, which play a key role in the spermatogenesis (42, 43). Abnormal serum T/E_2_ ratios has been associated with decreased spermatogenesis (43–46). Accordingly, we found that food withdrawal induced a shift in steroid biosynthesis, since estradiol levels increased and testosterone levels decreased in testis compared to both controls, leading to a significant drop in the T/E_2_ ratio within the testis, accompanied by declined spermatogenesis. Although we found increased 17β-estradiol levels in fasted-rat testis, we did not observe increased levels in P450 aromatase protein expression. This may be due to increased activity of this enzyme under these conditions rather than increased expression. This hypothesis requires further investigation.

In both fed and fasted animals subjected to exercise, although testicular levels of 17β-estradiol resulted unchanged, the elevated levels of local testosterone significantly increased T/E_2_ ratio with respect to control and food withdrawal groups, respectively, improving spermatogenesis activity.

Testosterone levels in the serum closely reflected those observed in the testes, confirming previous studies using similar conditions (14, 33, 40, 47). In contrast, levels of 17β-estradiol in the serum did not parallel those observed within the testes, since food withdrawal significantly increased the levels of this hormone in the testis but not in the serum. This result implies that although P450 aromatase is also expressed in various tissues (48), 17β-estradiol synthesis during food withdrawal may be stimulated prevalently in testis. This underlines the importance of measuring intratesticular testosterone and 17β-estradiol levels in order to verify if spermatogenesis is influenced by the experimental interventions. Nevertheless, serum T/E_2_ correlated with testis T/E_2_ ratio, confirming that exercise rescues the decline in food withdrawal-induced spermatogenesis.

In conclusion, this study highlights that food withdrawal or exercise alone, as well as food withdrawal combined with exercise affected spermatogenesis activity, through the modulation of steroidogenic pathway. In addition, we provide consistent evidence supporting the beneficial effects of mild exercise in preventing and/or counteracting impaired testis function as a result of reduced caloric intake.

## Data Availability Statement

The datasets generated for this study are available on request to the corresponding author.

## Ethics Statement

The animal study was reviewed and approved by Committee on the Ethics of Animal Experiments of the University of Campania Luigi Vanvitelli and the Italian Ministry of Health (authorization 704/2016 PR pursuant to article 31 of legislative decree 26/2014).

## Author Contributions

AS and AG co-wrote the paper and performed experiments. SF, FD, and RS performed experiments. MD supervised the experiments. GC, AL, and PL designed the experimental concepts and edited the paper. PL edited the final version of the paper. All authors approved with the content of the paper.

## Conflict of Interest

The authors declare that the research was conducted in the absence of any commercial or financial relationships that could be construed as a potential conflict of interest.
